# Personalized therapy: CNS HGNET-BCOR responsiveness to arsenic trioxide combined with radiotherapy

**DOI:** 10.18632/oncotarget.23174

**Published:** 2017-12-11

**Authors:** Claudia Paret, Alexandra Russo, Henrike Otto, Arnulf Mayer, Sebastian Zahnreich, Wolfgang Wagner, David Samuel, David Scharnhorst, David A. Solomon, Girish Dhall, Kenneth Wong, Hannah Bender, Francesca Alt, Arthur Wingerter, Marie A. Neu, Olaf Beck, Dirk Prawitt, Stefan Eder, Nicole Henninger, Khalifa El Malki, Nadine Lehmann, Nora Backes, Lea Roth, Larissa Seidmann, Clemens Sommer, Marc A. Brockmann, Gundula Staatz, Heinz Schmidberger, Jörg Faber

**Affiliations:** ^1^ Section of Pediatric Oncology, Children's Hospital, University Medical Center of The Johannes Gutenberg University Mainz, Mainz, Germany; ^2^ Department of Radiation Oncology and Radiation Therapy, University Medical Center of The Johannes Gutenberg University Mainz, Mainz, Germany; ^3^ Section of Pediatric Neurosurgery, Department of Neurosurgery, University Medical Center of The Johannes Gutenberg University Mainz, Mainz, Germany; ^4^ Department of Oncology, Valley Children’s Hospital, Madera, California, USA; ^5^ Department of Pathology, Valley Children’s Hospital, Madera, California, USA; ^6^ Division of Neuropathology, University of California, San Francisco, California, USA; ^7^ Division of Hematology, Oncology and Blood & Marrow Transplantation, Children’s Hospital Los Angeles, Los Angeles, California, USA; ^8^ Children's Center for Cancer and Blood Diseases, Children's Hospital Los Angeles, Los Angeles, California, USA; ^9^ Center for Pediatrics and Adolescent Medicine, Children's Hospital, University Medical Center of The Johannes Gutenberg University Mainz, Mainz, Germany; ^10^ Institute of Pathology, University Medical Center of The Johannes Gutenberg University Mainz, Mainz, Germany; ^11^ Division of Neuropathology, University Medical Center of The Johannes Gutenberg University Mainz, Mainz, Germany; ^12^ Department of Neuroradiology, University Medical Center of The Johannes Gutenberg University Mainz, Mainz, Germany; ^13^ Section of Pediatric Radiology, Department of Diagnostic and Interventional Radiology, University Medical Center of The Johannes Gutenberg University Mainz, Mainz, Germany; ^14^ University Cancer Center of The Johannes Gutenberg University, Mainz, Germany

**Keywords:** ATO, HGNET-BCOR, radiation, liquid biopsy, targeted therapy

## Abstract

High-grade neuroepithelial tumor of the central nervous system with BCOR alteration (HGNET-BCOR) is a rare, highly malignant tumor. At the time of this publication, no standard protocol exists to treat this tumor entity. In this work, we tested the responsiveness of the primary culture PhKh1 derived from tumor tissue from a pediatric HGNET-BCOR patient (P1) to inhibitors of the Sonic hedgehog pathway combined with radiation. The SMO inhibitors vismodegib and itraconazole had low effect on the proliferation of the PhKh1 cells. However, the GLI inhibitor arsenic trioxide reduced the expression of GLI target genes in the PhKh1 cells and in combination with radiotherapy significantly decreased their clonogenic potential. PhKh1 cells resistant to arsenic trioxide were characterized by the overexpression of molecular chaperones. We combined arsenic trioxide and radiation in the relapse therapy protocol of P1, achieving complete remission after seven weeks. Clinical remission lasted for six months, when P1 developed systemic metastases. Meanwhile, an increase in the concentration of circulating tumor DNA carrying a BCOR internal tandem duplication was observed. Molecular characterization of a second patient (P2) was also performed. In P2, we detected a larger tandem duplication and greater activation of the Sonic hedgehog pathway than in P1. These findings suggest that combining arsenic trioxide with radiotherapy may represent a new therapeutic approach. Moreover, peripheral blood analysis for circulating tumor DNA could help in the early detection of systemic metastases.

## INTRODUCTION

High-grade neuroepithelial tumor of the central nervous system with BCOR gene alteration (CNS HGNET-BCOR) is a rare entity described first in 2016 affecting particularly children [1]. HGNET-BCOR represents 3% of tumors with an institutional diagnosis of “CNS-PNET” according to the old World Health Organization diagnostic lexicon. HGNET-BCOR is characterized by somatic internal tandem duplication (ITD) in the C-terminus of BCL-6 co-repressor (BCOR) associated with an upregulation of *BCOR* expression. The same duplication has been also described in clear cell sarcoma of the kidney [2], soft tissue undifferentiated round cell sarcoma of infancy (URCSI) and primitive myxoid mesenchymal tumor of infancy (PMMTI) [3]. Preliminary survival data suggest that the CNS HGNET-BCOR entity has poor overall survival with most patients experiencing disease progression within the first year of diagnosis. Thus, new treatment options are highly warranted.

We and other have recently demonstrated the activation of the Sonic hedgehog (SHH) pathway in CNS HGNET-BCOR [1, 4]. The binding of the SHH ligand to the Patched-1 (PTCH1) receptor relieves smoothened (SMO) inhibition, leading to activation of glioma-associated oncogene (GLI) transcription factors (GLI1-3). Activated GLIs accumulate in the nucleus and controls the transcription of SHH target genes supporting cell proliferation. While GLI activation may result from SHH ligand-induced signaling, there is mounting evidence for non-canonical signaling leading to the expression of GLI proteins [5].

The SMO and the GLI family of zinc-finger transcription factors are considered important targets for cancer therapeutics. The SMO inhibitor vismodegib has already been approved by the FDA for the treatment of basal cell carcinoma [6]. We previously have shown that a primary CNS HGNET-BCOR cell culture (PhKh1) is sensitive to arsenic trioxide (ATO) [4], a drug known to target the SHH pathway at the level of GLI proteins [7]. ATO is a FDA-approved drug used for the treatment of acute promyelocytic leukemia (APL), including pediatric patients [8].

In this study, we applied the concept of personalized therapy to a pediatric patient (P1) with a diagnosis of CNS HGNET-BCOR and upregulation of the SHH pathway. First, we tested several SHH pathway inhibitors on the tumor cells of the patient *in vitro*. Then, we showed a relationship between the dose of radiation and control of tumor growth. Based on these results, we developed a personalized treatment protocol for the patient which comprised of ATO and radiation and measured serum and cerebrospinal fluid (CSF) ATO concentrations. Finally, we monitored the tumor status through the analysis of the circulating DNA (ctDNA) in peripheral blood. We also report a second pediatric patient (P2) with CNS HGNET-BCOR where we were able to confirm activation of the SHH pathway at a higher level than in the first patient. The ITD of *BCOR* in P2 was also found to be longer compared to P1.

## RESULTS

### PhKh1 cells are more sensitive to GLI than to SMO inhibition

**Figure 1:**
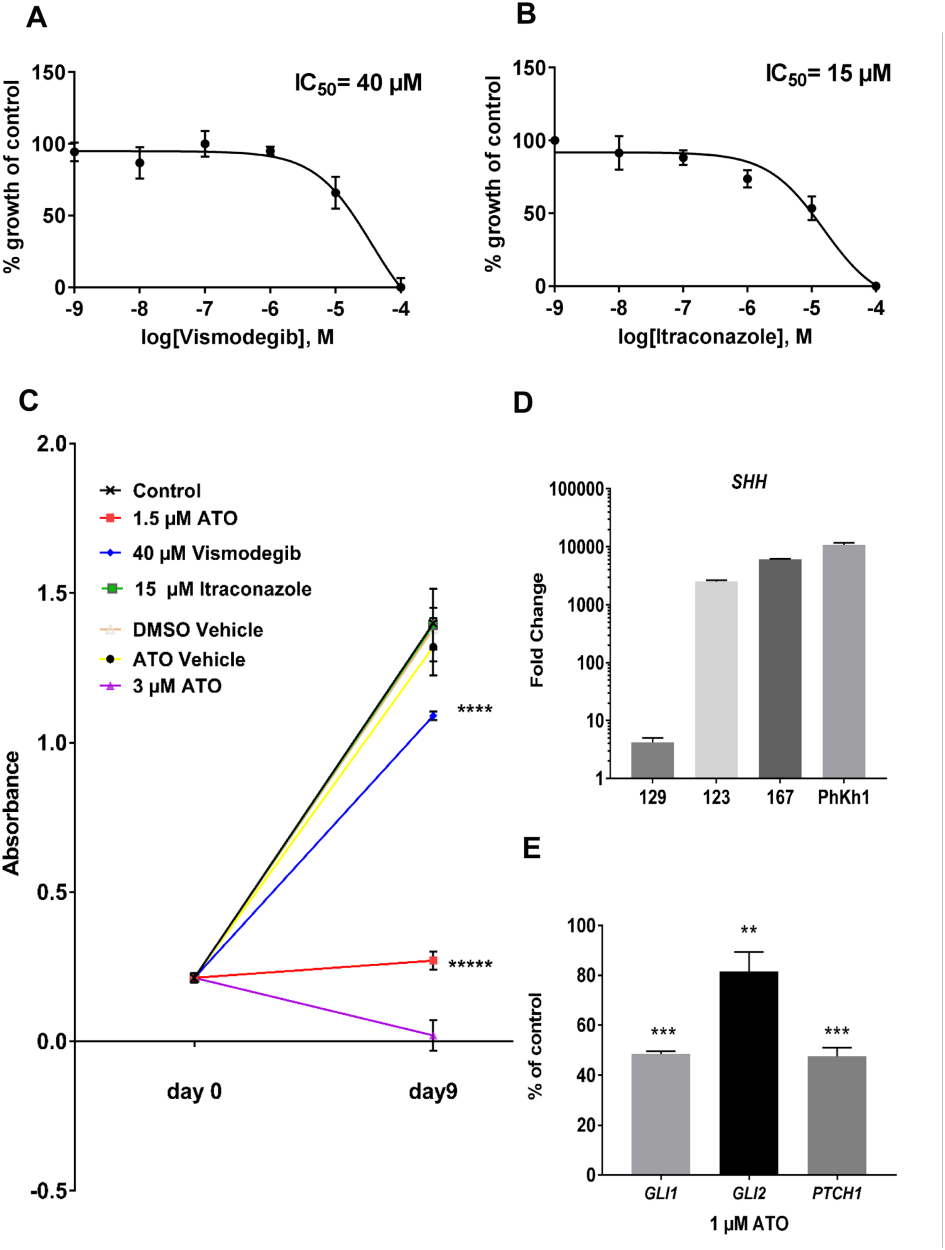
PhKh1 cells are more sensitive to GLI than to SMO inhibition **(A-B)** PhKh1 cells were treated with vismodegib or itraconazole at doses from 1 nM to 100 μM. The logarithm of the molarity is displayed on the X-axis. The percent of viable cells compared to the control treated with vehicle alone is shown on the Y-axis. The data were fitted to a sigmoidal dose-response curve using GraphPad software. A representative experiment of three independent experiments is shown. **(C)** The PhKh1 cells were grown for nine days in the presence of ATO, itraconazole, vismodegib or vehicle alone at the indicated concentrations. The absorbance after incubation with the WST-1 reagent is indicated. Statistics were performed using student’s t-test at day 9 compared to the control: ^****^p<0.0001, ^*****^p<0.00001. **(D)** The expression of the *SHH* ligand was analyzed by qRT-PCR in normal brain, the primary HGNET-BCOR tumor (no 123), a metastasis of HGNET-BCOR (no 167), a medulloblastoma of the WNT subtype (no 129) and the PhKh1 cells. The fold change of the expression with respect to normal brain is shown. **(E)** PhKh1 cells were incubated for 18 hours with 1 μM ATO or vehicle alone. After RNA extraction, qRT-PCR analysis of *GLI1 GLI2* and *PTCH1* was performed. The expression in the ATO-treated cells is shown as percent of the expression in the vehicle-treated cells. Expression analysis was done in triplicates. Statistics were performed using student’s t-test: ^***^ *p=0.001*, ^**^ *p=0.0056*.

We incubated the PhKh1 primary cells with different concentrations of vismodegib and itraconazole (Figure 1A and 1B), two drugs known to inhibit the SMO receptor with different mechanisms of action [9]. The IC_50_ of itraconazole was 15 μM and of vismodegib was 40 μM. Lower IC_50_ values of about 55 nM and 8 μM have been described for itraconazole- and vismodegib-sensitive cells, respectively [10, 11]. We previously have shown that PhKh1 cells are sensitive to the GLI inhibitor ATO with an IC_50_ of 1.5 μM. Whereas many traditional chemotherapeutics inhibit proliferation on the timeframe of hours or in a few days of treatment, targeted therapies that affect cancer-relevant pathways can require several days to impact cellular growth and survival. To study the long-term effect of GLI and SMO inhibition, we incubated the PhKh1 cells with ATO, vismodegib or itraconazole for nine days (Figure 1C). Itraconazole at 15 μM had no influence on long term cell proliferation. Vismodegib at 40 μM significantly reduced cell proliferation but not as efficiently as ATO at 1.5 μM. ATO at 3 μM completely inhibited cell growth. Thus, a lower concentration of ATO than of vismodegib is required to reduce cell growth.

Mutations in SMO have been described as a mechanism of resistance to the SMO inhibitor vismodegib [11]. We have previously described that the primary tumor and the metastases of P1 did not carry SMO missense mutations [4]. Here we extended the analysis to the PhKh1 primary cells. By Sanger sequencing we didn’t find any mutation changing the protein sequence of SMO (data not shown). These data suggest that the low sensitivity of the PhKh1 cells to SMO inhibition is not dependent on SMO alterations.

To exclude that a loss of the expression of the *SHH* ligand in the cell culture system could account for the reduced sensitivity to SMO specific inhibition, we analyzed the expression of the *SHH* ligand by qRT-PCR in P1. The *SHH* ligand was highly expressed in the primary tumor, in a metastasis and in the PhKh1 cells, but not in a medulloblastoma tissue of the WNT subtype (Figure 1D). These data indicate that the expression of the *SHH* ligand is maintained in the cell culture system.

To further understand the mechanism of action of ATO, we analyzed the expression of the GLI target genes *GLI1*, *GLI2* and *PTCH1* after incubation with 1 μM ATO (Figure 1E). The expression of *GLI1*, *GLI2* and *PTCH1* was circa 49%, 81%, and 46% of the control, respectively. These results suggest that ATO decreases the expression of target genes of the SHH pathway, thus inhibiting cell proliferation.

In summary, these results suggest a partially SMO-independent GLI transcriptional activity that can be inhibited by ATO *in vitro*. The inhibition of the SHH pathway can be monitored by the reduction in the expression of the SHH target genes and correlates with the anti-proliferative potential of ATO in long-term experiments. An ATO concentration of 3 μM was required to fully block the growth of the PhKh1 cells in long-term experiments. This concentration is achievable in clinical setting [12].

### Resistance to ATO is characterized by the up-regulation of transcripts coding for stress proteins

If PhKh1 cells were grown under 1 μM ATO, resistant clones inevitably grew. To understand the mechanisms of resistance, we performed transcriptome analysis of the PhKh1 cells grown under 1 μM ATO or under vehicle alone. Eight genes were highly upregulated in the resistance cells compared to control (Table 1). Of these, *ANXA1*, *CLU, SLC17A1* and *RCAN1* encode for stress proteins [13–16]. ANXA1, ANXA3, CLU and CTGF are associated with drug resistance [17–20]. Interestingly, upregulation of cell migration-inducing protein (CEMIP) has been associated with enhanced cell migration [21]. These data indicate that ATO induces the expression of proteins that may allow the cellular recovery from stress, thus restoring protein homeostasis and promoting cell survival. Co-targeting these stress-induced survival pathways may better manipulate cancer cell sensitivity to ATO therapy.

**Table 1:** Transcripts upregulated in the ATO resistant cells

Gene	Entrez gene name	Expr fold change	TPM_control	TPM_ATO	Location
CTGF	connective tissue growth factor	98.309	5.803	570.486	Extracellular Space
ANXA1	annexin A1	63.838	5.192	331.465	Plasma Membrane
RCAN1	regulator of calcineurin 1	61.117	25.688	1569.973	Nucleus
ANXA3	annexin A3	55.225	1.181	65.216	Cytoplasm
SMN2	survival of motor neuron 1. telomeric	35.726	1.000	35.726	Nucleus
SLC7A11	solute carrier family 7 member 11	23.848	1.000	23.848	Plasma Membrane
CEMIP	cell migration inducing hyaluronan binding protein	22.876	4.328	99.020	Cytoplasm
CLU	clusterin	20.445	9.577	176.646	Cytoplasm

The Expr fold change indicates the ratio between the TPMs of PhKh1 cells grown under ATO (TPM_ATO) and the TPMs of the PhKh1 cells grown under vehicle alone (TPM_control). The location of the corresponding proteins are also indicated.

### ATO causes additive cytotoxicity to ionizing radiation in PhKh1 cells

**Figure 2:**
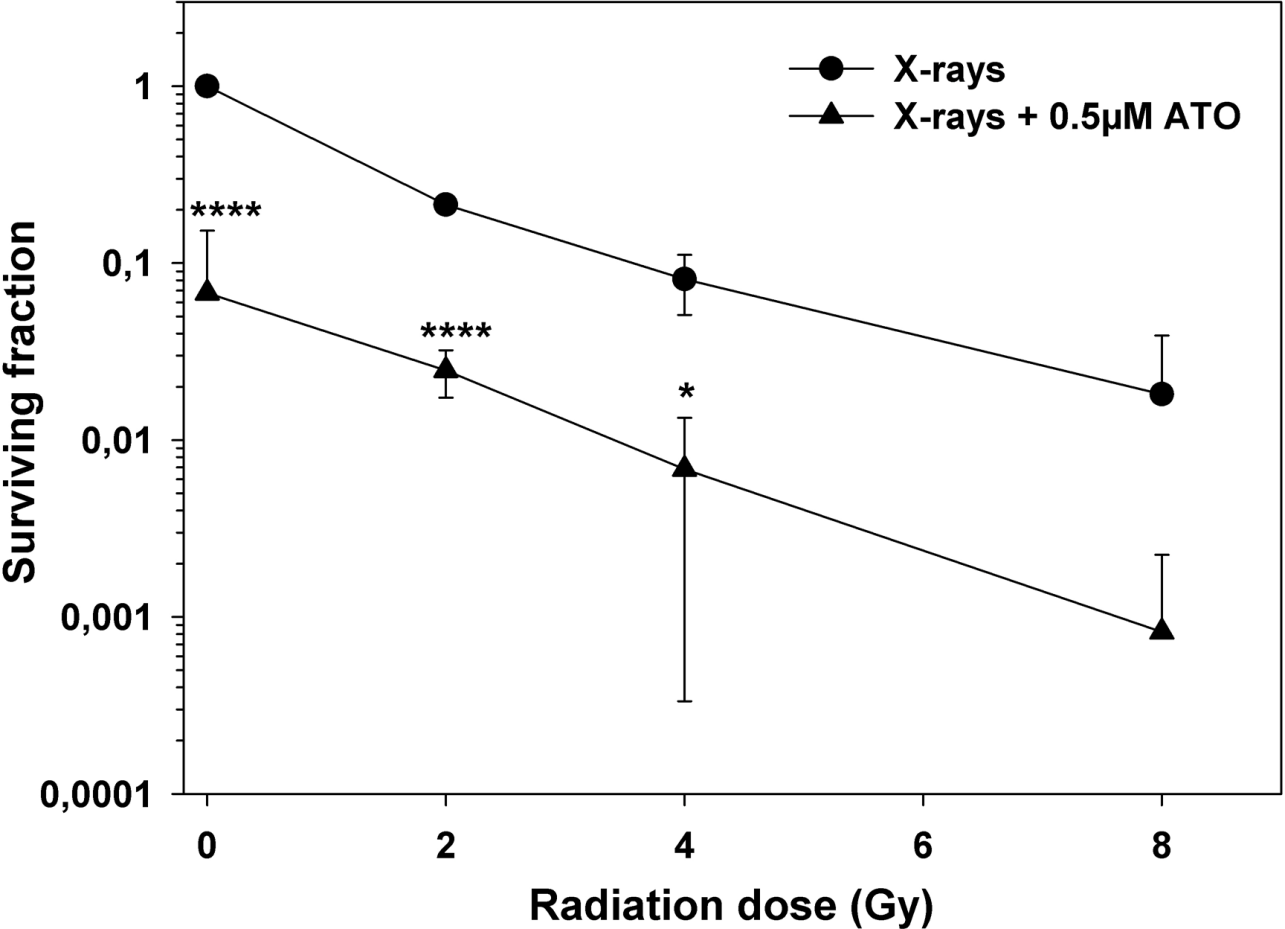
Additive cytotoxicity of radiation and ATO in PhKh1 cells Dose-response survival curves of PhKh1 cells after exposure to X-rays with or without ATO. Data are shown as the mean ± standard deviation from three independent experiments. Lines are drawn to guide the eye. All data were normalized to non-irradiated cells without ATO. Statistics were performed using student’s t-test: ^*^p<0.05, ^****^p<0.0001.

As fractionated radiation therapy is central to the treatment of high-grade central nervous system tumors, we measured the *in vitro* clonogenic survival of PhKh1 cells after X-ray exposure with or without concomitant ATO treatment. Figure 2 shows the respective radiation dose-response survival curves. Treatment of PhKh1 cells with 0.5 μM ATO alone reduced the clonogenic survival to about 10% of untreated cells. Treatment just with ionizing radiation reduced the viability of PhKh1 cells in a dose-dependent manner. Concomitant radiation and ATO significantly reduced the clonogenic survival compared to radiation alone by additive cytotoxicity.

### HGNET-BCOR response to irradiation

**Figure 3:**
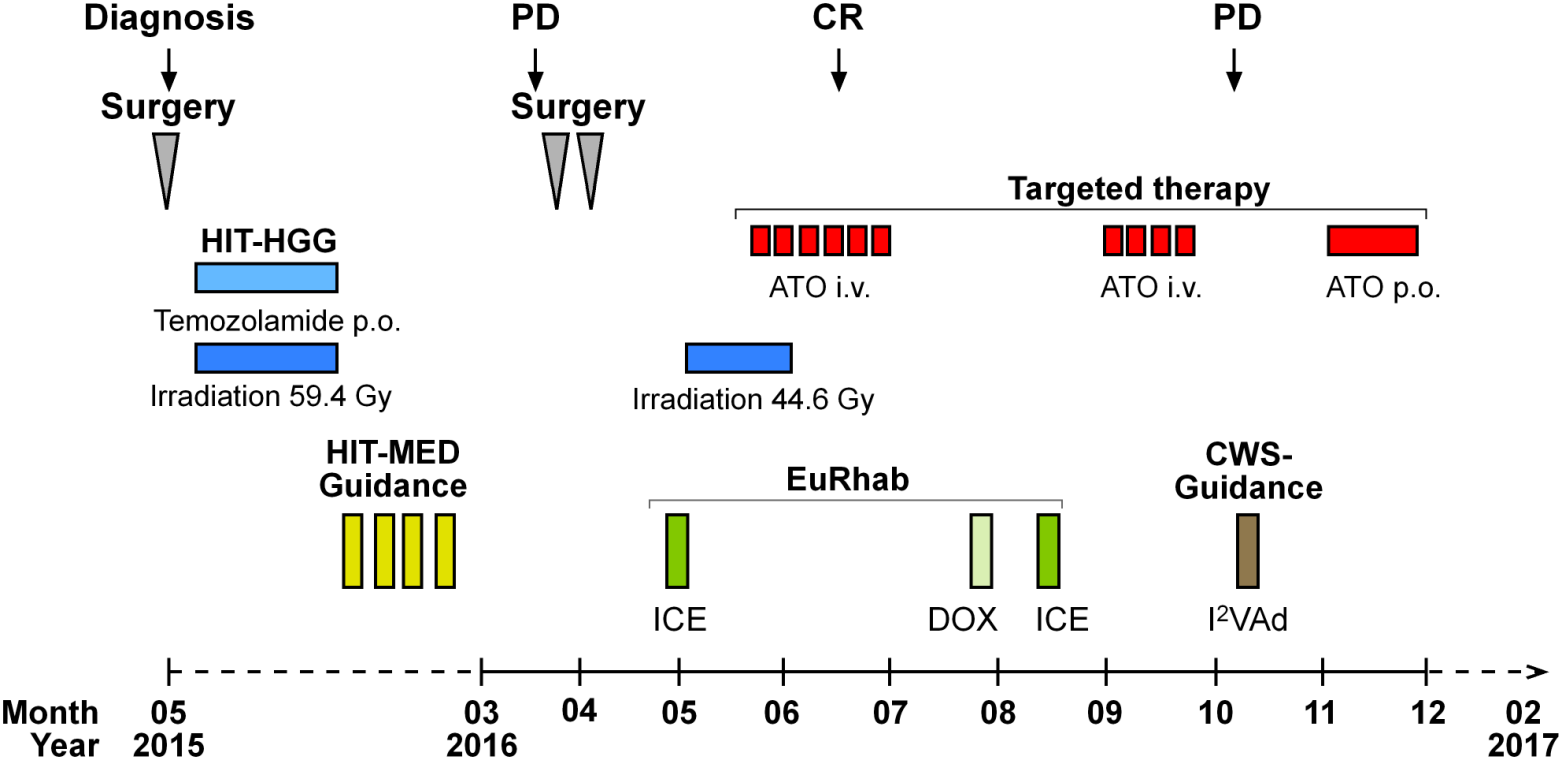
Therapy of P1 With a presumed diagnosis of a malignant glioma, we initiated treatment according to the HIT-HGG protocol (cranial irradiation with 59.4 Gy in 30 fractions with concomitant oral temozolamide chemotherapy). Due to the following diagnosis of a “primitive neuroectodermal tumor with WNT-like subtype” we added 4 cycles of chemotherapy with vincristine, cisplatin and CCNU according to the HIT-Med protocol. The relapse treatment protocol combined conventional radio-chemotherapy with an individualized therapy approach. The back-bone chemotherapy, included elements from pediatric rhabdoid and soft-tissue sarcoma protocol (EURHAB; CWS-Register “SoTiSaR”, Soft Tissue Sarcoma Registry). After surgical resection, the sites of the metastatic skull lesions were irradiated with 44 Gy (5 x 2 Gy/week). ATO was administered to coincide with the last two weeks of radiation. We conducted two cycles of intravenous (i.v.) ATO, given five days per week for six weeks in the first cycle (ATO I) and for four weeks in the second cycle (ATO II) with an interval of eight weeks in between. Following the diagnosis of progressive, systemic disease, we switched to an oral (p.o.) ATO formulation for four weeks. PD and CR were assessed by MRI. PD = progressive disease; CR = clinical remission; ATO = arsenic trioxide; ICE = ifosfamide, carboplatin, etoposide; DOX = doxorubicin; I^2^VAd = ifosfamide, vincristine, dactinomycin C.

**Figure 4:**
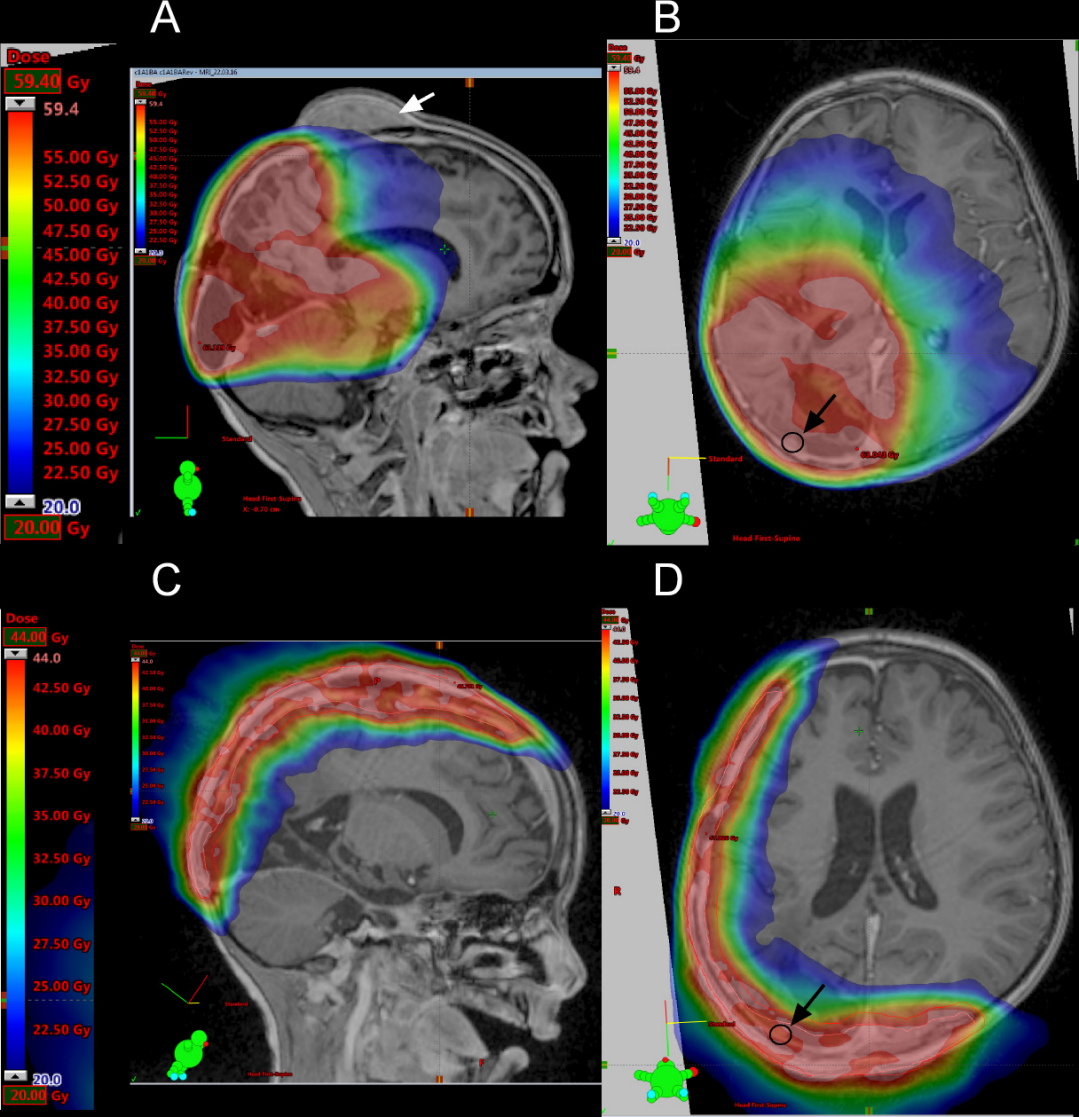
Irradiation fields used in the treatment protocol **(A-B)** MRI showing the irradiation field used in the first line therapy. The intensity of the irradiation field is shown in color. The gradation is from 20 Gy (blue) to 59.4 Gy (red). **(C-D)** MRI showing the irradiation field used in the targeted therapy. The gradation is from 20 Gy (blue) to 44 Gy (red). The white arrow indicates a skull metastasis, the dark circle and the dark arrow indicate a dural lesion.

The primary tumor of P1 was localized to the right parieto-occipital lobe [4]. The first line therapy of P1 included irradiation based on the HIT-HGG protocol and maintenance therapy based on the HIT-MED protocol (Figure 3) but the P1 developed three metastatic lesions to the skull during maintenance chemotherapy. All three metastatic lesions were located close to the area of the former resection cavity, suggesting that the tumor cells were displaced from the primary tumor during the initial resection. Detailed review of the irradiation field indicated that all three metastases arose outside of the high-dose irradiation area (Figure 4A). In parallel with the skull metastases, a newly formed dural lesion was also detected at the supero-lateral margin of the former resection cavity to the right parieto-occipital lobe (Figure 4B, circle). The dural lesion received 59.4 Gy in the first irradiation. We used this metastatic dural lesion as a measurable disease to monitor subsequent response to the personalize target therapy described below.

### Personalized therapy of P1

Macroscopic complete resection of the skull metastases was performed. Local and reference pathology laboratory analysis of these tissues samples revealed the same tumor entity. Our relapse treatment protocol combined conventional radio-chemotherapy with an individualized therapy approach based on our molecular characterization of the tumor (Figure 3). As a back-bone of chemotherapy, we integrated treatment elements from pediatric rhabdoid and soft-tissue sarcoma protocol (EURHAB; CWS-Register “SoTiSaR”, Soft Tissue Sarcoma Registry) based on the scientific rationale that GLI overexpression has been also detected in pediatric rhabdoid tumors as well as in several sarcoma cell types [22–24]. Chemotherapy incorporated ifosfamide, carboplatin, etoposide regimen (ICE), ifosfamide, vincristine, dactinomycin C (I^2^VAd) regimen and doxorubicin (DOX).

Due to the noted radiosensitivity of the tumor, re-irradiation with 44 Gy (5 x 2 Gy/week) of the former position of the metastatic skull lesions was performed (high parietal/frontal and occipital) (Figure 4C and 4D). Re-irradiation was done with 3 cm safety margin and included the newly formed dural lesion which was re-irradiated to 44 Gy (Figure 4D, circle). ATO was administered to coincide with the last two weeks of radiation. To reach therapeutically meaningful concentrations, we chose a dose 0.05 mg/kg higher than the dose recommended for the treatment of pediatric patients with promyelocytic leukemia [8]. We conducted two cycles of intravenous ATO (0.2 mg/kg), given five days per week for six weeks in the first cycle (ATO I) and for four weeks in the second cycle (ATO II) with an interval of eight weeks in between. To avoid electrolyte disturbances serum electrolytes were closely monitored throughout treatment with ATO and potassium supplements given as needed. Electrocardiograms were monitored before the start of ATO and then weekly during treatment to evaluate for possible QT prolongation, a known side effect of ATO. The concomitant treatment with ATO and radiotherapy did not increase adverse effects in P1, in keeping with previous reports in pediatric patients [25].

**Figure 5:**
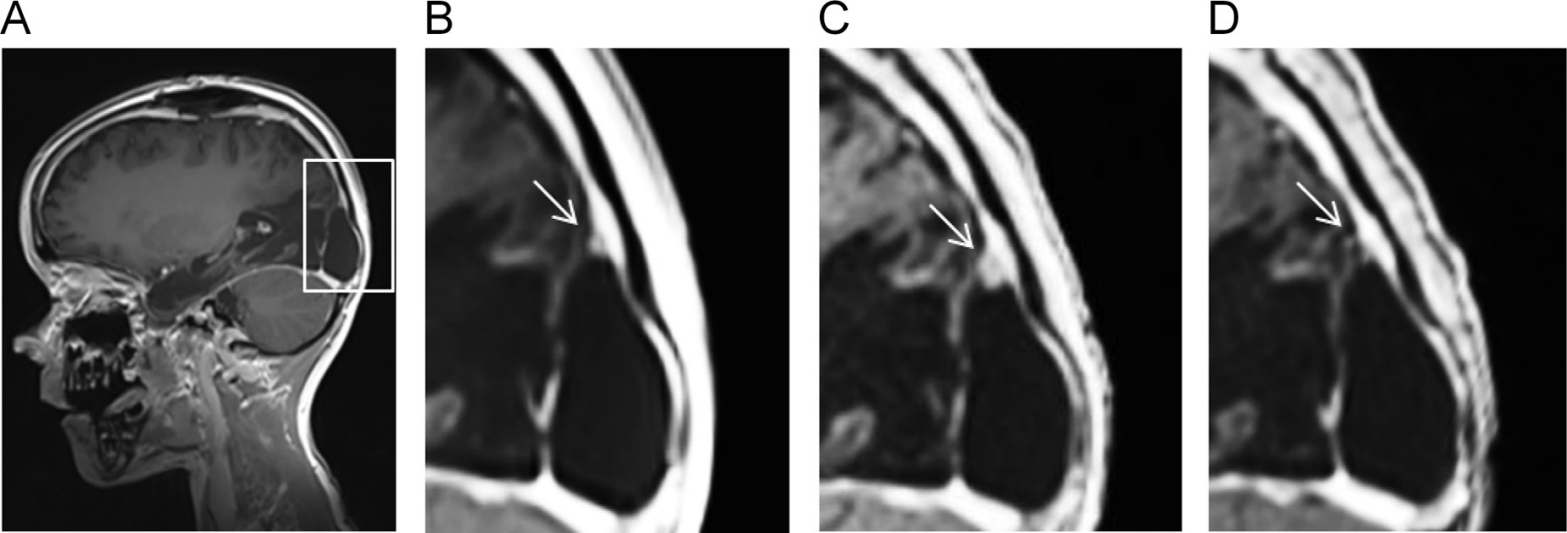
The target lesion is sensitive to the targeted therapy MRI showing the dural lesion developed under the first line therapy **(A-B)**, before the start of the targeted therapy **(C)** and at the time of CR **(D)**.

Radiographic follow-up using magnetic resonance imaging (MRI) revealed a continuous treatment response with the patient achieving complete remission (CR) with no measurable disease activity seven weeks after the initiation of relapse therapy (calculated starting from the first ICE block) including four weeks of targeted therapy with intravenous ATO (Figure 5A-5D). CR was confirmed by a further MRI nearly two months later. Five months after the initiation of the relapse therapy (and 6 months after the resection of the metastases) the patient developed cranial and extra cranial metastases in lung, liver, spinal column and bones (Supplementary Figure 1). Notably, the former site of relapse in the skull was not involved.

Following the diagnosis of progressive, systemic disease, we switched to an oral ATO formulation in order to forgo hospitalization of the patient. To increase the plasma arsenic concentration, we extended the oral formula dosage of ATO to 0.3 mg/kg (ATO III). The oral ATO formulation was prepared in our hospital pharmacy based on the publication of Kumana et al. [26]. The administration of parenteral ATO is well established in the therapy of acute promyelocytic leukemia (APL) and the oral formulation of ATO appears to have a comparable efficiency [26, 27] although, the clinical use of oral ATO in pediatric patients is less common. ATO was well tolerated intravenously and orally. No adverse cardiac effects, in particular, QT prolongation [28] or any skin reactions were observed. Furthermore, P1 did not develop any electrolyte disturbances with oral substitution of potassium. The oral ATO was given continuously for a period of four weeks. Due to the highly malignant nature of the disease, P1 died 22 months after initial diagnosis and 10 months after relapse.

### The concentration of arsenic in plasma and CSF samples of P1 didn’t reach the IC_50_

The total amount of arsenic at the end of each cycle was 48.3 μg/l (ATO I) and 46.9 μg/l (ATO II) in plasma and 9.1 μg/l (ATO I) and 6.5 μg/l (ATO II) in CSF. After 2 weeks of ATO III, the concentration was 45.3 μg/l in plasma and 9.7 μg/l in CSF. In accordance with several reports [29], we observed a linear correlation (r =0.854; p <0.05) between the two compartments, with CSF levels at 16.5% of the plasma level. This was equivalent with a plasma/CSF ratio around 6:1. Regarding the systemic bioavailability of ATO, parenteral and enteral administration provided comparable concentrations in the plasma and CSF compartements. These results coincide with findings from adult APL patients treated with intravenous and oral ATO [26]. In conclusion, the maximal arsenic concentration achieved in P1 was of 48.3 μg/l in plasma and of 9.7 μg/l in CSF corresponding to a maximum arsenic concentration of 645 nM and 129 nM respectively, a concentration that was below the IC_50_.

### The *BCOR* ITD is maintained in the systemic metastases

**Figure 6:**
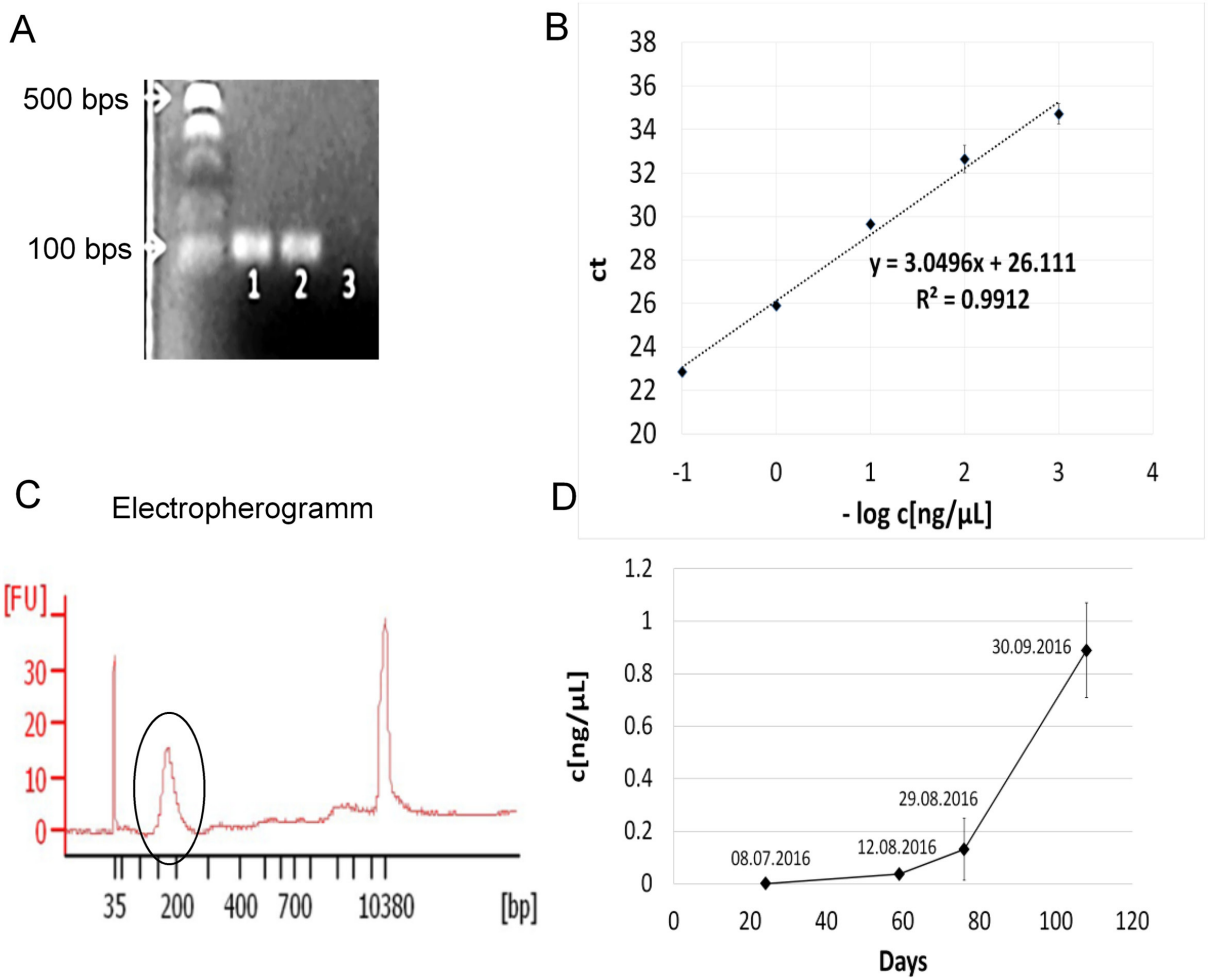
The *BCOR* ITD is maintained in the systemic metastases of P1 **(A)** *BCOR* ITD specific primers were used for PCR analysis of the genomic DNA extracted from the primary tumor (lane 1), a metastasis (lane 2) and the blood (lane 3) of P1. The expected product of *BCOR* ITD is 117 bps. **(B)** The genomic DNA extracted from the primary tumor was diluted at different concentrations. The –log of the concentration in ng/μl is shown on the x-axis. The threshold cycle (ct) is shown on the y axis. The slope of the standard curve was used to calculate the efficiency of the primers. **(C)** The size of the isolated ctDNA was analyzed using a Bioanalyzer. The y axis shows the signal intensity (FU) and the x axis the size distribution in bps. The circle indicates the purified ctDNA. **(D)** The concentration of the ctDNA was calculated based on the standard curve in B and reported as ng of ctDNA per μl of purified ctDNA (ng/μl) on the y axis. The x axis report the days of the plasma collection with respect to the time of the complete remission as assessed by MRI (x=0). To facilitate the comparison with the sketch in Figure 3, the datum of the plasma collection is also reported.

Because tissue biopsies of the systemic metastases at the time of the second relapse were not performed, we used peripheral blood to monitor the tumor status in P1, especially the persistence of the *BCOR* ITD under the targeted therapy. First we established primers for the detection of the *BCOR* ITD. The sequence of the *BCOR* ITD of P1 has been already described [4]. The primers detected the *BCOR* ITD in the genomic DNA extracted from the primary tumor and a metastasis from P1 but not the *BCOR* wild type (wt) in the genomic DNA extracted from the blood of P1 (Figure 6A) and had an efficiency of 100% (Figure 6B). We extracted the ctDNA from four samples of peripheral blood at different points of time after the detection of CR by MRI. One sample was collected 24 days after the radiographic detection of CR and three further samples were collected prior to the radiographic appearance of systemic metastatic disease. The concentration of the ctDNA ranged from 0.338 ng/μl to 5.820 ng/μl. The ctDNA was highly fragmented as expected [30] with a median size of about 160 bps (Figure 6C). Twenty-four days after the CR, the concentration of the *BCOR* ITD was at the limit of detection of our assay (Figure 6D). After circa two months, a clear increase in the concentration of the *BCOR* ITD was detectable and reached the maximum four days before the radiographic detection of the systemic metastases by MRI (Figure 6D, day 108). These data indicate that the *BCOR* ITD is still present in the lesions that developed after the systemic spread of the disease and that the liquid biopsy of peripheral blood represents a powerful method for the early detection of systemic metastasis in these patients.

### The SHH pathway is upregulated in a second CNS HGNET-BCOR patient (P2)

**Figure 7:**
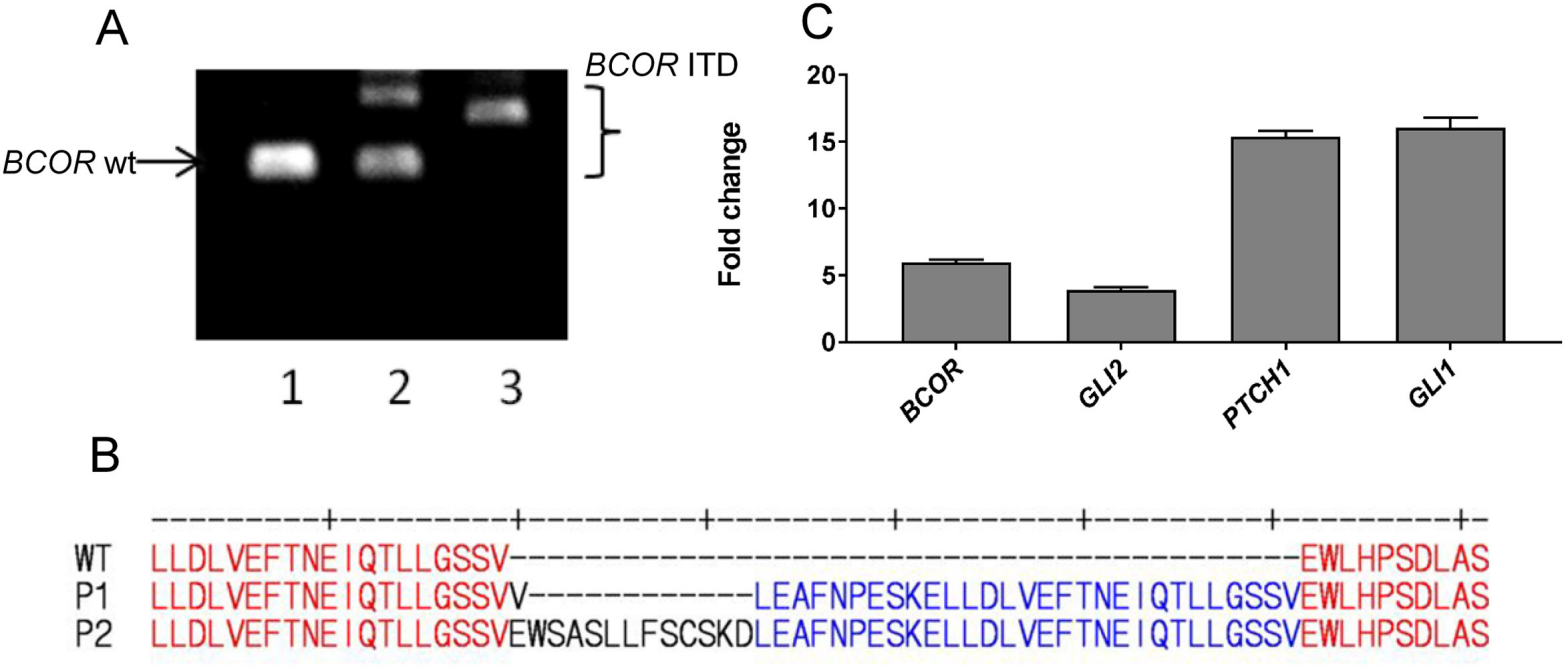
The SHH pathway is upregulated in an additional CNS HGNET-BCOR case **(A)** The sequence of exon 15 of *BCOR* was analyzed by PCR in the genomic DNA extracted from the blood of P1 (lane 1), the primary tumor of P2 (lane 2) or from the primary tumor of P1 (lane 3). The sizes of the wt *BCOR* and of the *BCOR* ITDs are indicated. **(B)** Protein sequence of the BCOR allele of P2 carrying the ITD compared to the wild type BCOR and to the BCOR ITD detected in P1. **(C)** qRT-PCR analysis was performed using primers recognizing *BCOR*, *GLI2, PTCH1* or *GLI1*. After normalization to the housekeeping gene *HPRT1*, the fold change of the expression of P2 with respect to P1 was calculated. Expression analysis was done in triplicates. Expression analysis was done on RNA extracted from FFPE material.

CNS HGNET-BCOR is characterized by tandem duplication in exon 15 of the *BCOR* gene. This tandem duplication can be of different sizes. We analyzed exon 15 in the genomic DNA extracted from the blood and the primary tumor of P1 and from the primary tumor of P2 by PCR (Figure 7A). Only the *BCOR* wt could be detected in the blood of P1, only the *BCOR* ITD could be detected in the tumor of P1 and the wt and the *BCOR* ITD could be both detected in the tumor of P2. This can be explained by the fact that *BCOR* is located on the X chromosome and therefore male individuals carry only one allele. We cloned and sequenced the *BCOR* ITD in P2 revealing an internal tandem duplication of 126 nucleotides (42 amino acids) (Figure 7B). If the presence of the wt *BCOR* allele in female patients can influence the severity of the disease remains to be elucidate. The insertion in P2 is larger than that detected in P1 and is one of the largest described at the time of this publication [1]. The ITD of P2 and P1 are both localized in a domain required for the binding of BCOR to a Polycomb proteins complex [31]. We validated the upregulation of the SHH pathway in P2 by qRT-PCR. Expression of *BCOR* in P2 was even stronger than in P1 (Figure 7C). To functionally validate the activation of the SHH pathway, we analyzed the expression of *GLI1*, *GLI2* and *PTCH1*. All three genes were highly expressed in P2, even stronger than in P1. These results confirm the assumption that the upregulation of the SHH pathway is a common feature of HGNET-BCOR. However, while the expression of GLI2 was confirmed at the protein level in tumor material extracted from P1 (Supplementary Figure 2), no fresh frozen material was available to perform the analysis in P2 and we cannot conclude if the higher RNA expression in P2 corresponds to a higher protein expression.

Treatment of P2 was given according to the Children’s Oncology Group ACNS 0334 protocol, in an attempt to delay or avoid radiotherapy [32–34]. Therapy consisted of three induction cycles of chemotherapy, including high-dose methotrexate, and three consolidation cycles of myeloablative chemotherapy with autologous hematopoietic stem cell rescue. However, as more information on the propensity of this tumor entity to relapse became available, a decision to treat with low-dose craniospinal irradiation (18 Gy) and tumor bed boost (54 Gy) was made. Treatment details of P2 can be found in the Supplementary Materials. To date, 20 months from diagnosis and 10 months from completing treatment, the patient remains in clinical and radiological remission.

## DISCUSSION

HGNET-BCOR is a recently described rare tumor entity of the central nervous system for which optimal treatment protocols are yet to be defined. Because patients with HGNET-BCOR were previously diagnosed as other entities, several treatment protocols have been attempted. To date, protocols for the treatment of CNS-PNET, glioblastoma and ependymoma have been used, all of which have included high doses of chemotherapy and radiation. According to the available data on follow up, no protocol has been successful in the treatment of this tumor entity with most patients developing progressive disease [1]. A recent clinicopathologic and molecular characterization of 3 cases of HGNET-BCOR [35] confirmed the difficulty encountered in controlling this tumor entity using surgical resection of the primary tumor followed by radiation and chemotherapy. Here we show that the combination of conventional sarcoma-based chemotherapy, irradiation and ATO administration led to complete remission after extra-cerebral relapse with six months of progression-free survival.

Available microarray data suggest that the upregulation of the SHH pathway is a common feature of HGNET-BCOR. We have previously validated the activation of the SHH pathway in P1 [4] and in this work in P2. Thus, a targeted therapy against this pathway is of interest for HGNET-BCOR patients. The therapeutic effect of the SMO inhibitor itraconazole has been recently discussed in one HGNET-BCOR female pediatric patient [35]. Unfortunately no *in vitro* data on the sensitivity of the HGNET-BCOR cells to itraconazole were available for that patient. Moreover, SMO inhibition causes permanent defects in bone structure in young mice, and its use in young children has to be carefully considered with respect to long-term toxicities [36]. Our data suggest a partially SMO-independent GLI transcriptional activity in the PhKh1 cells which has been described in other tumor entities. A possible mechanism for the SMO-independent activation of GLI could rely on the presence of the ITD. BCOR enhances BCL6-mediated transcriptional repression by interacting with histone deacetylases (HDAC) [37]. Moreover, BCOR forms a complex with Polycomb proteins and can act as an epigenetic repressor [38]. BCOR wt can repress the expression of *GLI1* and *GLI2* [39]. The ITD is localized in a region required for the binding to the Polycomb complex and required for the full activity of BCOR [31]. Thus the ITD may interfere with the repressor function of BCOR leading to the upregulation of *GLI1* and *GLI2*. This hypothesis has to be validated but it is in line with the observed upregulation of *GLI* transcripts in clear cell sarcoma of the kidney which also express the *BCOR* ITD [2]. Thus, targeting the SHH pathway downstream of SMO at the level of the GLI transcripts using ATO is a particularly attractive approach in HGNET-BCOR. Here we show that ATO reduced the transcription of GLI target genes in a primary culture of HGNET-BCOR. In APL, ATO triggers the degradation of the PML–RARα fusion protein, probably by binding to a cysteine-rich region [40]. However, no cysteine-rich regions are created by the presence of the ITD. Thus, the effect of ATO on GLI in HGNET-BCOR is probably direct and not via BCOR. Indeed ATO has been shown to reduce GLI activity by direct binding to GLI proteins [7]. Moreover, the upregulation of GLI itself seemed to be responsible for the ineffectiveness of conventional chemotherapy [41, 42] and earlier studies demonstrated additive effects of ATO with conventional chemotherapeutic agents such as cisplatin, adriamycin, and etoposide [43, 44]. The down-regulation of GLI transcription factors via ATO to sensitize HGNET-BCOR cells to conventional chemotherapy, particularly to the sarcoma-based chemotherapy applied in the relapse protocol warrants further investigation.

The radiosensitivity of HGNET-BCOR is suggested by the growth of the skull metastases outside of the high dosage area. The relevance of postoperative craniospinal irradiation in patients suffering from CNS HGNET-BCOR has been recently discussed [35]. However, irradiation alone was not able to control the progression of the disease in P1. Indeed, the target lesion developed in a region that was irradiated with 59.4 Gy during the irradiation of the primary tumor suggesting the necessity to add radiation sensitizers, like ATO, to the treatment protocol. Recent reports have shown synergistic radio-sensitizing effects by concomitant treatment with ionizing radiation and ATO in a variety of tumor cell lines *in vitro* [45–47] as well as in tumor xenografts [48, 49]. Our work indicates additive cytotoxic effects of radiation and ATO on the clonogenic survival of PhKh1 cells *in vitro* at an ATO concentration lower than in the available literature mentioned above. Our finding of additive cytotoxicity of radiation and ATO in a HGNET-BCOR cell-strain offers a rationale for concomitant treatment regimens to achieve a higher tumor response and reduce radiation dosage during whole-brain radiotherapy to lower the risks for radiation-related late adverse effects in pediatric patients. Considering acute and long-term toxicity of arsenic agents, we observed no severe adverse effects during and after the six-month period with a cumulative dose of 18.4 mg/kg. Notably, the good tolerance of continuous treatment with oral ATO together with no need to hospitalize the patient besides regular monitoring of the arsenic concentration might be beneficial for pediatric patients.

ATO at a concentration of 1.5 μM effectively reduced the growth of the PhKh1 cells, and the growth was completed inhibited if the cells were cultivated in the presence of 3 μM ATO. A peak level of 5.54 μM to 7.30 μM plasma arsenic can be achieved in APL patients with an acceptable safety profile [12]. Arsenic can penetrate the CNS of APL patients with a mean concentration of 199 nM [50]. This concentration can be increased to 0.5 μM by the addition of mannitol [51]. Moreover, higher concentration of ATO is known to accumulate in tumor tissues compared to normal brain and the total concentration of ATO in the plasma or CSF may not reflect the true concentration in tumor tissue [52]. Importantly, 1.0 μM ATO showed little influence on the viability of cortical neurons [53] while we observed a radio-sensitizing of ATO already at a concentration of 0.5 μM. Thus, therapeutically meaningful and safe concentrations of arsenic in plasma and CNS can be achieved. With our protocol, we achieved a maximum arsenic concentration of 645 nM in plasma and of 129 nM in CSF, a concentration that was below the IC_50_. The low levels of arsenic achieved in this study could explain why the patient progressed under the targeted therapy after the initial response. Of course, no conclusion can be drawn from only one case but efforts could be undertaken in future cases to increase the ATO concentration for example by a daily ATO administration, possibly orally and to understand the pharmacokinetic and pharmacodynamic of ATO in HGNET-BCOR patients.

Tumor evolution escaping conventional and targeted therapy is a major limitation for the successful treatment of cancer. We were able to detect the *BCOR* ITD in the ctDNA isolated from the peripheral blood of the patient P1, at the time systemic metastases developed, demonstrating that despite the use of aggressive conventional and targeted therapy, the tumor can still carry the alteration detected at the time of the first diagnosis. This finding underlines the relevance of the ITD in the biology of this tumor. Because HGNET-BCOR patients can develop systemic metastases, peripheral blood analysis for circulating *BCOR*-ITD DNA could help in the early identification of high-risk patients.

The PhKh1 primary tumor cells resistant to ATO overexpressed transcripts coding for the molecular chaperones CLU and ANXA1. Chaperones are expressed in response to chemotherapy and play a crucial role in maintaining the stability and activity of numerous signaling proteins required in the tumor development, including the processes of metastases formation. Therefore chaperons are considered as important targets in cancer treatment [54]. Down-regulating CLU or ANXA1 expression may represent an attractive therapeutic strategy to enhance the effect of ATO treatment. Second-generation antisense oligodeoxynucleotide designed to inhibit CLU are currently in late-stage clinical development [55]. The resistant cells also expressed CEMIP, a protein associated with cancer cell survival, migration and invasion. However, CEMIP has been reported to play roles in cancer cell proliferation and invasion either negatively or positively [56] and its role in the tumor progression of HGNET-BCOR remains to be clarified. Because tumor material of the metastases developed under the targeted therapy was not available, we were not able to proof if the molecular chaperones and CEMIP were overexpressed *in vivo* and contributed in fact to the development of the metastases.

P2 remained free of disease 20 months after the first diagnosis. The presence of the wt *BCOR* allele in the P2 patient (female) could be related to the better prognosis compared to the P1 patient (male). Because *BCOR* is on the X chromosome, male patients have only the copy with the ITD while female patients can still carry the wt allele, as described in the two patients in our study. It would be interesting to analyze if female patients with a relapse loss the wt *BCOR* allele. Intriguingly, all tumor entities carrying the *BCOR* ITD show a male predominance [35]. However, there are not enough published outcome data to conclude on a possible gender-related prognosis in HGNET-BCOR.

In conclusion, our results present evidence that the targeting of GLI transcripts via ATO, in combination with radiotherapy in addition to conventional back-bone chemotherapy is a promising approach that warrants validation in a clinical study.

## MATERIALS AND METHODS

### Patient primary tissue samples, CSF, peripheral blood and cell lines

Clinical history of P1, tumor samples and the short-term primary cell culture (PhKh1) isolated from this patient have already been described [4]. Briefly, sample 123 was derived from the primary tumor of P1, sample 167 from a metastasis from the same patient and sample 129 was isolated from a medulloblastoma of the WNT subtype. To monitor the concentration of arsenic under targeted therapy with ATO, plasma and CSF from P1 were collected at different time points. Surplus plasma was used for the extraction of ctDNA. This study was performed in agreement with the declaration of Helsinki. In accordance with the ethics committee of Rhineland-Palatinate, the patient’s parents agreed with the scientific use of the surplus material. Informed consent of the patient’s parents for the targeted therapy was obtained. No further approval of the medical ethics committee was required as only one patient was involved.

P2 is a female infant who presented at the age of four years and nine months with a posterior fossa mass without leptomeningeal dissemination. The tumor was completely removed and after initial radiological and histological assessment was diagnosed as medulloblastoma. However, additional immunohistochemistry was performed during consultative pathologic review that showed negative synaptophysin staining and positive OLIG2 staining in a large fraction of the tumor cells, findings that are not typical for medulloblastoma. The CNS HGNET-BCOR diagnosis in P2 was based on targeted next-generation sequencing results from the UCSF500 Cancer Panel, which we have previously described [57], that detected an internal tandem duplication in exon 15 of the *BCOR* gene and absence of genetic alterations that are commonly seen in medulloblastomas. Formalin-fixed and paraffin-embedded (FFPE) material derived from the primary tumor was used for the analysis. Consent to publish details of the therapy and the results of the molecular analysis was obtained from the parents and approved by the patient’s hospital Institutional Review Board (IRB).

### Nucleic acid extraction

DNA was extracted using the Gentra Puregene Blood Kit or the QIAamp DNA FFPE (Qiagen, Hilden, Germany). RNA extraction was performed using the RNeasy Mini Kit or the RNeasy FFPE Kit for FFPE material (Qiagen). RNA was converted to cDNA using PrimeScript RT Reagent Kit with gDNA Eraser (Takara Bio Europe, Saint-Germain-en-Laye, France). Quality control was performed using a 2100 Bioanalyzer (Agilent Technologies, Waldbronn, Germany). Due to expected low quality of the RNA extracted from FFPE, the protocol for cDNA synthesis was changed. Instead of utilizing the RT Primer Mix that is included in the kit, we used gene-specific reverse primers and the reaction samples were incubated for 60 minutes at 42 degrees Celsius instead of 15 minutes at 37 degrees Celsius. ctDNA was isolated from 1.5 ml plasma using the QIAamp Circulating Nucleic Acid Kit (Qiagen). ctDNA was eluted in 20 μl water and analyzed using a 2100 Bioanalyzer. The *BCOR* ITD was detected with following primers: 5’- GGCTCCTCTGTAGTCCTGGA and 5’- GGGGTGGAGCCACTCTACA.

### RT-PCR and qRT-PCR

RNA was converted to cDNA by using PrimeScript RT Reagent Kit with gDNA Eraser (Takara Bio Europe, Saint-Germain-en-Laye, France). qRT-PCR was performed using the LightCycler 480 II Detection System and Software (Applied Biosystems, Darmstadt, Germany) with KAPA SYBR FAST LightCycler 480 Kit (PeqLab, Erlangen, Germany). The sequence of the primers can be found in the Supplementary Materials. After normalization to the housekeeping gene *HPRT1*, the relative quantification value was expressed as 2^−ΔΔCt^. The calibrator was calculated as the maximal number of cycles used in the PCR (40) minus the mean of the *HPRT1* Ct values, resulting in a value of 19.

### DNA sequencing and TA cloning

The coding exons of *SMO* were amplified by PCR using 50 ng of DNA. The primers were as described in [4]. The region containing the *BCOR* ITD was amplified using primers 5’-GGAAATTGTCACCATTGCAGAGG and 5’-TGTACATGGTGGGTCCAGCT. Sanger Sequencing was performed as previously described [4]. TA cloning was performed by TA Cloning^®^ Kit with pCR™2.1 Vector and One Shot^®^ TOP10F' Chemically Competent E. coli (Thermo Scientific, Dreieich, Germany). The TA cloning method takes advantage of the terminal transferase activity of some DNA polymerases adding a 3'-A overhang to each end of the PCR product. This makes it possible to clone this PCR product directly into a linearized cloning vector with single, 3'-T overhangs. Clones were analyzed by Sanger sequencing.

### Cellular proliferation assays

Itraconazole and vismodegib (both Selleckchem, Houston, USA) were dissolved in DMSO at a concentration of 20 mM and 50 mM, respectively. ATO (Sigma-Aldrich, Taufkirchen, Germany) was prepared as described previously [4]. Cells were plated in triplicates at a density of 5,000 cells/well in a 96-well plate. Itraconazole and vismodegib at varying concentrations or vehicle alone were added to the cells. Viable cells were quantified after three days using the cell proliferation reagent WST-1 (Roche, Mannheim, Germany). Dose-response curves were plotted to determine the half-maximal inhibitory concentration (IC_50_) using the GraphPad Prism v.5 (GraphPad Software, San Diego CA, USA). For long-term experiments, 5,000 cells were plated in triplicates and incubated with different concentrations of itraconazole, vismodegib, ATO or vehicle alone.

### Clonogenic assay

Survival curves of PhKh1 cells were generated after X-ray exposure or after concomitant treatment with radiation and 0.5 μM ATO using an in-vitro clonogenic assay [58]. Cells were irradiated with doses of 0, 2, 4 and 8 Gy and seeded at densities of 5 × 10^3^, 1 × 10^4^, 2 × 10^4^ and 3 × 10^4^ per well, respectively. Three independent experiments were performed. ATO was added after 24 hours and cells were exposed to X-rays (140 kV) at room temperature using the D3150 X-ray Therapy System (Gulmay Medical Ltd., UK) at a dose rate of 3.62 Gy/min or were mock treated, i.e., kept for the same time in the radiation device control room. Cells were incubated for 14 days to allow colony formation, stained with 0.25% crystal violet and colonies containing ≥ 50 cells were counted as survivors. Plating efficiency (PE) was assessed as the ratio of the number of colonies counted as survivors to the number of cells seeded. The surviving fraction (SF) was calculated as follows: SF = colonies counted/(cells seeded x PE), taking into consideration the individual PE.

### RNA sequencing

Libraries preparation, sequencing and Transcripts per Kilobase Million (TPM) calculation was as described previously [4]. To identify genes associated with ATO resistance, we calculated the ratio of the expression between the TPM of the PhKh1 cells grown under ATO and of the PhKh1 cells grown under the vehicle alone and selected genes with a fold change >20.

### Targeted therapy protocol

Relapse treatment protocol of P1 combined conventional radio-chemotherapy with an individualized therapy approach using intravenous and oral ATO. As back-bone chemotherapy, we integrated several treatment elements from pediatric rhabdoid and soft-tissue sarcoma protocols (EURHAB; CWS-Guidance). In accordance with our previous and current results from the PhKh1 cell cultures [4] we added ATO as individualized therapy approach to the relapse treatment protocol, with concurrent irradiation to the skull metastases (44 Gy, 5x2Gy/week). For details see the results section.

### Arsenic concentration

The total arsenic concentration in CSF and plasma was analyzed by a certified laboratory using inductively coupled plasma mass spectrometry (ICP-MS) (Medizinisches Labor Bremen, Bremen, Germany).

### Western blot analysis

Nuclear extracts were generated as previously described [4]. GLI2 antibody was from LifeSpan BioSciences (Eching, Germany) and Lamin B antibody from Cell Signaling.

## SUPPLEMENTARY MATERIALS FIGURES



## Abbreviations

APL: acute promyelocytic leukemia
ATO: arsenic trioxide
BCOR: BCL-6 co-repressor
CNS HGNET-BCOR: high-grade neuroepithelial tumor of the central nervous system with BCOR alteration
ct: threshold cycle
ctDNA: circulating DNA
CR: complete remission
CSF: cerebrospinal fluid
DOX: doxorubicin
ICE: ifosfamide, carboplatin, etoposide
ITD: internal tandem duplication
I^2^VAd: ifosfamide, vincristine, dactinomycin C
PD: progressive disease
PE: Plating efficiency
TPM: Transcripts per Kilobase Million
