# Supplementary material for: Personalized therapy: CNS HGNET-BCOR responsiveness to arsenic trioxide combined with radiotherapy
Source: Oncotarget. 2017 Dec 11;8(69):114210–25. doi: 10.18632/oncotarget.23174 (PMC5768397; doi:10.18632/oncotarget.23174)
Supplement: Supplementary file 1 [file oncotarget-08-114210-s001.pdf]

Personalized therapy: CNS HGNET-BCOR responsiveness to arsenic trioxide combined with radiotherapy

SUPPLEMENTARY MATERIALS

Primers used in the work

Following primers were used: *BCOR*: 5’- GGCA GCTCTGTTTGTGAACC and 5’- CCTGAGCCACAG ATACTTGG; *GLI1*: 5’- AGCTTGTCCCACACCGGTAC and 5’- GAGGATGCTCCATTCTCTGGTG; *GLI2*: 5‘-TCCACACACGCGGAACACCA and 5’ CAGCTGGC TCAGCATGGTCA, *PTCH1*: 5’- CAGCTCCAACTGAG GGTGAT and 5’- AATGAACTGCTGTCCTGGCAC, *SHH:* 5’- GTGAAAGCAGGCAAGGAAAGGA and 5’- AAACTCTTGGCTCCGTCAACC; *HPRT1*: 5′-TGACA CTGGCAAAACAATGCA and 5′-GGTCCTTTTCACCA GCAAGCT.

Therapy of P2

Following the diagnosis of CNS HGNET-BCOR in patient 2 (P2), treatment was commenced according to the Children’s Oncology Group ACNS 0334 protocol. Three cycles of Induction chemotherapy, each beginning with high-dose intravenous (i.v.) methotrexate (400 mg/kg) was given over four hours after vincristine (0.05 mg/kg) i.v. as a 5 minute infusion on day 1 with hydration prior to and after the methotrexate, until serum methotrexate levels were reduced to less than 0.01 µmol/L. This was followed by i.v. cyclophosfamide (60 mg/kg) with i.v. etoposide (2.5 mg/kg) daily for two days followed by i.v. cisplatin (3.5 mg/kg) for one day. Induction chemotherapy cycles were given every 21 days following the recovery of the absolute neutrophil count (ANC) to greater than 1000/µL and a platelet count of greater than 100,000/µL. Peripheral blood stem cell collection was performed after the first induction cycle with granulocyte colony stimulating factor (G-CSF) and 30×10^6 CD34+ cells were collected, divided into 5 bags, and frozen at -80°C for later use. Following induction chemotherapy, three tandem consolidation (high-dose) chemotherapy cycles with autologous stem cell rescue (ASCR) given every 28 days and consisted of i.v. carboplatin (17 mg/kg) and i.v. thiotepa (10 mg/kg) daily for two days. This was followed by a day of rest before infusion of previously harvested autologous hematopoietic stem cells at a dose of 6×10^6 CD34 positive cells per kg during each of the three consolidation cycles. Subcutaneous (s.c.) G-CSF was given daily at a dose of 5 µg/kg, starting 24 hours after the infusion of stem cells and continued until the ANC surpassed 2,000/µL on three consecutive days. Both induction and consolidation chemotherapy with AuHSCR chemotherapy was well tolerated with the expected complications of myelosuppression, mucositis, fever and pancytopenia. Because of the previously described overall poor prognosis of CNS HGNET-BCOR, the prior experience with patient 1 (P1) and *in vitro* the radiosensitivity of the tumor reported here, pre-emptive low-dose radiotherapy was given. The craniospinal dose consisted of ten fractions of 180 cGy for a total craniospinal dose of 1800 cGy followed by a boost of 3600 cGy delivered in twenty fractions for a cumulative dose of 5400 cGy to the posterior fossa tumor bed. To date, five months following the completion of therapy and 15 months after the initial diagnosis, the patient remains in clinical and radiological remission.

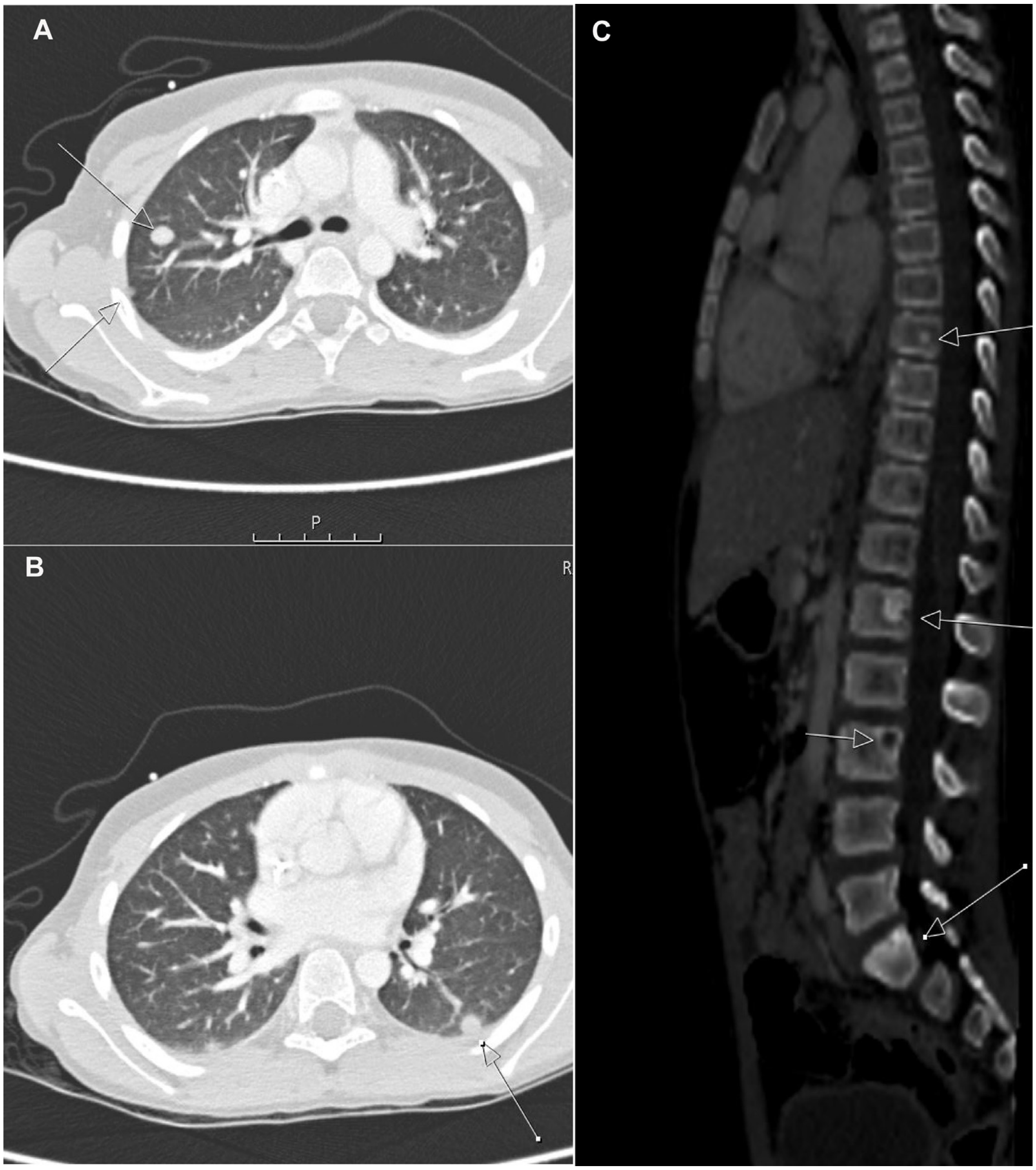

Supplementary Figure 1: Systemic metastases of P1 detected by computer tomography (CT). The arrows indicate metastases in the lung **(A** and **B)** and in the spinal column **(C)**.

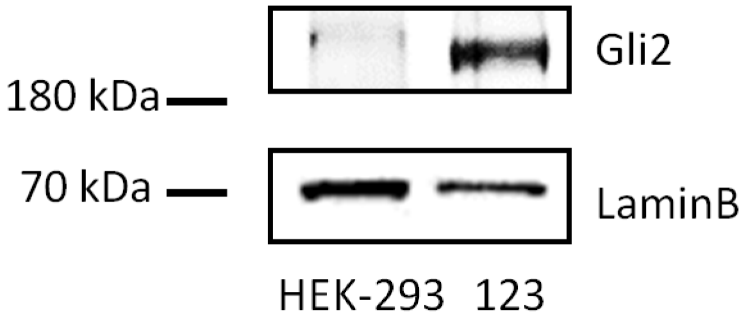

Supplementary Figure 2: GLI2 is expressed at the protein level. Nuclear fractions isolated from the HEK-293 cell line and the primary tumor of P1 (no 123) were analyzed by western blot with a GLI2 specific antibody or an antibody against Lamin B. A band of about 180 kDa as expected based on previous publications [59] was detected in the HGNET-BCOR tumor but not in HEK-293 cells which express low amount of GLI2 (qRT-PCR, data not shown).
